# Serum lipids as an indicator for the alteration of liver function in patients with hepatitis B

**DOI:** 10.1186/s12944-018-0683-y

**Published:** 2018-03-05

**Authors:** Sadia Qamar Arain, Farah Naz Talpur, Naseem Aslam Channa, Muhammad Shahbaz Ali, Hassan Imran Afridi

**Affiliations:** 1 National Centre of Excellence in Analytical Chemistry, University of Sindh, Jamshoro, 76080 Pakistan; 2 Institute of Biochemistry University of Sindh, Jamshoro, Pakistan; 3 Chulalongkorn University, Pathuwan, Bangkok, Thailand

**Keywords:** Hypolipidemia, GC-fid, Liver

## Abstract

**Background:**

Hepatitis B virus (HBV) exerts an intense impact on host lipid metabolism. Hence the aim of present study is to determine metabolic derangement that occurred in subjects suffering from hepatitis B patients.

**Methods:**

The fasting blood samples were collected from hepatitis B patients (*n* = 50) attended in Taluka hospital TandoAdam, Sindh with age and gender matched controls (n = 50). Serum lipid profile and fatty acid (FA) composition were analyzed by micro-lab and gas chromatography.

**Results:**

The hepatitis B patients have significantly lower level (*p* < 0.01) of lipid profile including total cholesterol (TC), triacylglyceride (TAG), high density lipoprotein-C (HDL-C) very low density lipoprotein-cholesterol (VLDL-C), low density lipoprotein-cholesterol (LDL-C), and total lipid (TL) in comparison to controls, indicating hypolipidemia in patients. The result of total FA composition of HBV patients in comparison to controls reveal that myristic, palmitic, docosahexaenoic acids were significantly (*p* < 0.05) higher, while linoleic, eicosatrienoic, arachidonic, eicosapentaenoic acids were lower in HBV patients in comparison to controls. The elongase, ∆5 and ∆6-desaturase enzymes activities were found lower, while ∆9-desaturase activity was higher in hepatitis B patients as compared to controls, which indicates the impaired lipid metabolism.

**Conclusion:**

The serum saturated fatty acid (SFA) and monounsaturated fatty acid (MUFA) were increased while polyunsaturated fatty acid (PUFA) was reduced in both total and free form in hepatitis B patients due to altered activities of enzyme desaturases with impaired PUFA metabolism and non-enzymatic oxidation**.**

**Electronic supplementary material:**

The online version of this article (10.1186/s12944-018-0683-y) contains supplementary material, which is available to authorized users.

## Background

Hepatitis B is a substantial health concern disturbing people all around the world. The World Health Organization in 2015 estimated that 240 million people have been already suffering in the chronic stage of hepatitis B virus (HBV) infection Worldwide, and 780,000 people lost their lives every year from hepatitis B [1]. Pakistan is having high dominance of HBV infection in all provinces. Approximately 9 million people are infected with HBV and its infection is growing evermore in our country day by day [2].

Ress et al., in 2016 [3] has reported that diseases agents interfere with the lipid metabolism by altering the liver function, which results in gathering of lipid drops in the liver cells that may cause in hepatic steatosis which results in defective secretion of very low density lipoprotein (VLDL) and alteration in the beta-oxidation. The fatty acid (FA) synthesis pathways are also affected by increased production of non-esterified FA, which is a major causing factor of fatty liver patients with HBV and hepatitis C virus (HCV) [4]. Over the past decade, HBV and altered plasma metabolites and their impaired functions become the subject of interest for biomedical researchers. The liver is an essential regulating organ plays a critical role by modulating exogenous and endogenous lipid metabolism. It is involved in the generating and reprocessing of lipid metabolites including lipoproteins such as high density lipoprotein-C (HDL-C), low density lipoprotein-C (LDL-C), total plasma cholesterol and triacylglyceride (TAG). The circulating level of those metabolites in plasma depends upon the functional capability of liver cells and tissues. Chronic HBV infection is directly affecting the functional capability of liver cells [5]. Mild to severe liver disrupting reasons such as chronic HBV infection could probably affect directly or indirectly on the levels of these lipid substrates in the plasma [6].

The fatty acids analysis in serum provided direct evidence on the fatty acids synthesis in the liver [7]. The saturated fatty acid (SFA), such as palmitic acid and yields of its conversion in to monounsaturated fatty acids (MUFAs), and polyunsaturated fatty acids (PUFAs) from the n-3 and n-6 families are connected through the metabolic activity of the liver [8]. Unger., in 2003 [9] has reported that free fatty acids (FFAs) are important mediators of lipotoxicity, they act as possible cellular toxins and leads to the lipid over-accumulation. When lipids are over-accumulated in non-adipose tissue, they may enter into non-oxidative deleterious pathways leading to cell injury and death. Qualitative and quantitative analyses of the FFA in serum provide an ancillary source of evidence on the synthesis of FA in the liver [10].

This information proposes that a relationship between metabolic alterations and HBV infection. Hence, the present study was undertaken to explore the changes and metabolic derangement in lipid metabolism of total lipid (TL), total cholesterol (TC), TAG, HDL–C, LDL–C, VLDL–C, total fatty acid (TFA) and FFA as reflected in the circulation of hepatitis B patients to compare this with that of healthy controls.

## Methods

**Fig. 1:**
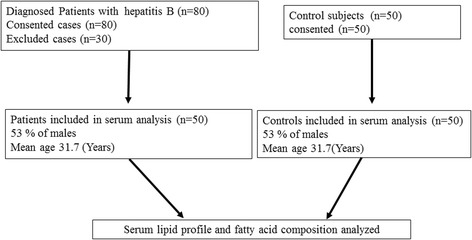
The flow chart of study

Hepatitis B is a severe form of viral hepatitis transmitted in infected blood, causing fever, debility, and jaundice. The patients attended in Taluka hospital TandoAdam with fever, headache, malaise, anorexia, nausea, vomiting, diarrhea, and abdominal pain and jaundice, their blood samples were subjected to enzyme-linked immunosorbent assay (ELISA) and alanine aminotransferase (ALT) test. The HBV positive patients with elevated ALT levels were further confirmed by Polymerase chain reaction (PCR) HBV deoxyribonucleic acid (DNA) active replication (> 1.30–8.23 log IU/mL reactive and < 1.3 log IU/mL non-reactive) and enrolled in the study. Age and gender matched controls with the negative history of hepatitis B (confirmed by ELISA test) were also included in the study. The flow chart of study is presented in Fig. 1.

### Sample collection and analysis

5 ml intravenous blood samples from HBV-positive patients and healthy controls (HBV-negative) were collected after 14 h overnight fasting. Serum was separated and stored at − 40 °C until analyzed for lipid profile and fatty acids by micro-lab 300 and gas chromatograph 8700 (Perkin–Elmer Ltd). FAs were analyzed as TFA and FFA. TFA, as well as FFA contents of the samples, were analyzed as per reported method [11]. Peaks were identified by authentic standards supplied by Fluka Chemika (Buchs, Switzerland). Analytical grade reagents and solvents were utilized throughout the study. The peak area was used to calculate FA composition as a relative ratio of the total FA.

### The desaturase and elongase enzymes activities calculation

Desaturase and elongase activities were calculated as the ratio of product to the precursor of an individual FA in serum (g/100 g) according to the following expression:

Δ-9 desaturase (D9D) = 16:1/16:0 and 18:1/18:0;

Δ-6 desaturase (D6D) = 18:3/18:2.

Δ-5 desaturase (D5D) = 20:4/20:3; and elongase = 18:0/16:0. [12].

### Model for end stage liver disease (MELD) calculation

MELD score was calculated by using the base line characteristics through “Online UNOS MELD calculator” [13]:

$$ {\displaystyle \begin{array}{l}\mathrm{MELD}\ \mathrm{Score}=0.957\times {\mathrm{Log}}_{\mathrm{e}}\left(\mathrm{creatinine}\ \left[\mathrm{mg}/\mathrm{dl}\right]\right)+0.378\\ {}\times {\mathrm{Log}}_{\mathrm{e}}\left(\mathrm{total}\ \mathrm{bilirubin}\ \left[\mathrm{mg}/\mathrm{dl}\right]\right)+1.120\\ {}\times {\mathrm{Log}}_{\mathrm{e}}\left(\mathrm{INR}\right)+0.64\end{array}} $$

### Statistical analysis

The data values are stated as mean with standard deviation. Student’s t-test /the Mann- Whitney U test was used for the relationship among the groups (controls vs. patients) with SPSS version 15 (SPSS Inc. Chicago, IL). Independent relationship of individual factor with Hepatitis B patients was evaluated by multivariable logistic regression analysis by the SAS statistical software (version 9.1; SAS Institute, Inc., Cary, North Carolina). Odds ratios and confidence intervals (CI) at 95% were designed to evaluate the risk factor for quartiles and continuous variables. Logistic-regression model was used to perform trends within each group. Quartile cut points with the lowest quartile were used as a reference to determine the division of the FA contents. The significant variations were observed when the *p* value was less than 0.05.

## Results

For the study, eighty hepatitis B patients consented; thirty patients were excluded by fulfilling criteria for exclusion as they were suffering from hepatitis co-infection, hypertension, diabetes, malnutrition, malabsorption, hyperthyroidism, renal failure, malignancy and immunoglobulin disorders. The blood specimens were also collected from fifty patients and fifty age and gender matched controls. The mean ± SD age of cases and controls was 31.7 ± 11.01 (age range17–60 years). Among the cases 53% were male.

### Base line characteristics

The base characteristics were collected during filling of the questionnaire and laboratory results of patients recorded. Table 1 shows the base line characteristics of hepatitis B patients including; random blood sugar, total protein, serum albumin, creatinine, total bilirubin, urea with in the normal limits and ALT level was increased in hepatitis B patients. The quartiles were calculated for base line characteristics of hepatitis B patients which reveals that cut-off for the first quartile is the 25th percentile, second quartile is the 50th percentile, which is the median and third quartile is the 75th percentile. It is evident that from the 25th percentile score majority of hepatitis B patients have serum albumin ≥3.4 g/dl. In addition score of 75th percentile indicated that most of hepatitis B patients possessed serum creatinine and total bilirubin below 1.0 mg/dl.

**Table 1:** Base line characteristics of Hepatitis B patients

Base line characteristics	Hepatitis B Patients (*n* = 50)	25th Percentile	50th Percentile	75th Percentile
Random Blood sugar (mg/dl)	115.50 (80–195)	99.2	106.0	125.0
Total Protein (g/dl)	6.52 (5.5–7.5)	6.0	6.5	7.1
Serum Albumin (g/dl)	4.10 (3.2–6.0)	3.4	4.0	4.6
Serum Creatinine (mg/dl)	0.76 (0.3–1.1)	0.6	0.8	1.0
Total bilirubin (mg/dl)	0.90 (0.5–1.2)	0.7	0.9	1.1
Urea (mg/dl)	24.82 (10–39)	20.0	25.0	30.0
SGPT or ALT (U/L)	76.23 (40 – 110)	66.0	75.0	88.7

### MELD score of hepatitis B patients

The MELD score was used to estimate relative disease severity and likely survival of patients awaiting liver transplantation. The hepatitis B patients MELD scores was 20 points showed 6.0% estimated 3 months mortality risk (Table 2).

**Table 2:** Model for end stage liver disease (MELD) in hepatitis B patients (n = 50)

Test	Normal Range (mg/dl)	Mean after results Interpretation	MELD Score
Creatinine	M: 0.6–1.1 FM: 0.7–1.3	1.5	MELD SCORE = 20
Bilirubin	M: 0.6–1.2 FM: 0.5–1.1	2.1	
INR	UNDER 1 .1	1.8	

### Lipid profile

The hepatitis B patients have significantly lower serum level (*p* < 0.001) of lipid profile including TC, VLDL-C, LDL-C, HDL-C, TAG and TL in comparison to controls (Table 3).

**Table 3:** Comparison of serum lipid profile of Hepatitis B patients with controls

Lipid profile	Controls (mg/dl) (n = 50)	Patients (mg/dl) (n = 50)
TC	152.40 ± 20.3	113.16 ± 17.1*
VLDL– C	23.50 ± 5.4	19.87 ± 3.6*
LDL– C	96.80 ± 15.4	87.10 ± 23.5*
HDL– C	46.74 ± 4.9	34.26 ± 9.8*
TAG	117.5 ± 27.0	99.36 ± 17.9*
TL	563.44 ± 50.9	478.04 ± 59.1*

Values are mean ± standard deviation*Different from Hepatitis B patients with healthy controls, *p* < 0.001 (t- test). Total cholesterol (TC), Total lipid (TL), Very low density lipoprotein-cholesterol (VLDL-C), low density lipoprotein-cholesterol (LDL-C), high density lipoprotein-cholesterol (HDL-C), and triacylglycerol (TAG)

### Fatty acid composition

The results of hepatitis B patients for serum TFA composition in comparison to controls revealed that myristic, palmitic, stearic, lignoceric, palmitoleic, oleic, docosenoic, docosahexaenoic acids were higher in hepatitis B patients and a significant difference was observed in myristic, palmitic, eicosapentaenoic acids. The linoleic, eicosatrienoic, arachidonic, eicosapentaenoic acids were significantly lower (< 0.05) in hepatitis B patients in comparison to control subjects. The α-linolenic was decreased but not significant. The PUFA: SFA ratio was reduced in HBV patients as compared to healthy subjects (Table 4).

**Table 4:** Comparison of serum total fatty acids composition in Hepatitis B patients and controls

Fatty acids	Controls (g/100 g) (n = 50)	Patients (g/100 g) (*n* = 50)
C - 14: 0	1.11 ± 0.8	2.04 ± 0.6*
C - 16: 0	21.67 ± 5.8	27.95 ± 3.3*
C - 18: 0	14.27 ± 4.9	13.27 ± 5.5
C - 20: 0	0.56 ± 0.9	0.43 ± 0.3
C – 24: 0	0.08 ± 0.2	0.10 ± 0.2
C - 14: 1	0.44 ± 0.8	0.46 ± 0.3
C - 16: 1	2.96 ± 2.0	3.55 ± 0.8
C - 18: 1	20.28 ± 4.4	21.64 ± 4.0
C - 22: 1	1.74 ± 1.3	1.92 ± 0.6
C – 24: 1	0.24 ± 0.6	0.09 ± 0.2
C - 18: 2	26.49 ± 5.0	21.48 ± 3.4*
C - 18: 3	0.90 ± 1.2	0.31 ± 0.3
C - 20: 3	0.90 ± 1.3	0.62 ± 0.3*
C - 20: 4	6.40 ± 1.9	3.72 ± 0.9*
C - 20: 5	1.08 ± 1.4	0.13 ± 0.2*
C - 22: 5	0.40 ± 0.3	0.79 ± 0.5*
C - 22: 6	0.45 ± 0.4	0.41 ± 0.4
PUFA: SFA	1.0	0.6

Values are mean ± standard deviation,*shows significant difference from Hepatitis B patients with comparison of healthy controls a *p* < 0.05 (t- test). myristic acid (C14:0), myristoleic acid (C14:1), palmitic acid (C16:0), palmitoleic acid (C16:1), stearic acid (C18:0), oleic acid (C18:1), linoleic acid (C18:2), α-linolenic acid (C18:3), arachidic acid (C20:0), eicosatrienoic acid (C-20:3), arachidonic acid (C20:4), eicosapentaenoic acid (EPA (C20:5), docosenoic acid (C22:1), docosahexaenoic acid (DHA (C22:6) lignoceric acid (C24:0), nervonic acid(C24:1)

**Fig. 2:**
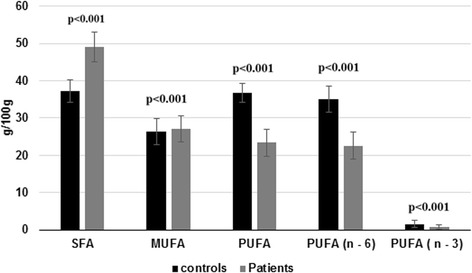
Comparison of SFA, MUFA, PUFA including n-3 and n-6 fatty acids in total FA composition of controls and hepatitis B patients

**Fig. 3:**
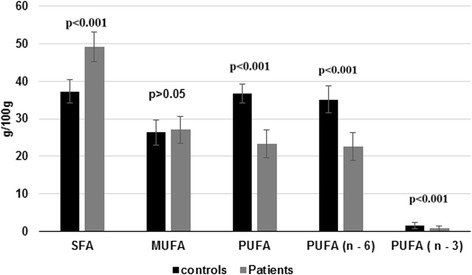
Comparison of SFA, MUFA, PUFA including n-3 and n-6 fatty acids in free FA composition of controls and hepatitis B patients

The serum SFA and MUFA was increased significantly (*p* < 0.001) in hepatitis B patients compared with normal controls and PUFA (n-3 and n-6) contents were significantly decreased (*p* < 0.05) in hepatitis B patients in comparison to controls both total and free form (Figs. 2 and 3).

The serum FFA profile of hepatitis B patient’s, showed significantly elevated (*p* < 0.05) level of palmitic and stearic acid. The arachidic, nervonic, linoleic acid, α-linolenic, eicosatrienoic, arachidonic, eicosapentaenoic and docosapentaenoic acids were significantly lower (< 0.05) in HBV patients in comparison to controls (Table 5). The serum SFA and MUFA was increased significantly (*p* < 0.05) in hepatitis B patients compared with normal controls and PUFA (n – 3 and n – 6) contents were significantly decreased (*p* < 0.05) in hepatitis B patients in comparison to controls (Table 4).

**Table 5:** Comparison of serum free fatty acids of Hepatitis B patients and controls

Fatty acids	Controls (g/100 g) (*n* = 50)	Patients (g/100 g) (n = 50)
C – 14:0	1.39 ± 0.6	1.61 ± 0.6
C - 16: 0	21.91 ± 4.3	28.70 ± 4.5*
C - 18: 0	13.33 ± 5.9	18.60 ± 6.4*
C - 20: 0	0.44 ± 0.8	0.08 ± 0.2*
C – 24: 0	0.13 ± 0.4	0.12 ± 0.2
C - 14: 1	0.19 ± 0.3	0.65 ± 0.3*
C - 16: 1	2.80 ± 1.2	3.39 ± 0.3
C - 18: 1	21.60 ± 4.5	21.52 ± 3.7
C - 22: 1	1.75 ± 1.2	1.37 ± 0.8
C – 24: 1	0.11 ± 0.1	0.11 ± 0.3
C - 18: 2	28.71 ± 5.1	19.50 ± 3.4*
C - 18: 3	0.60 ± 1.0	0.08 ± 0.2*
C - 20: 3	0.55 ± 0.8	0.02 ± 0.05*
C - 20: 4	5.67 ± 2.1	3.04 ± 1.1*
C - 20: 5	0.81 ± 1.3	0.03 ± 0.1*
C - 22: 5	0.22 ± 0.2	0.05 ± 0.1*
C - 22: 6	0.18 ± 0.2	0.63 ± 0.8*

Values are mean ± standard deviation,*shows significant difference from Hepatitis B patients with comparison of healthy controls a *p* < 0.05 (t-test). myristic acid (C14:0), myristoleic acid (C14:1), palmitic acid (C16:0), palmitoleic acid (C16:1), stearic acid (C18:0), oleic acid (C18:1), linoleic acid (C18:2), α-linolenic acid (C18:3), arachidic acid (C20:0), eicosatrienoic acid (C-20:3), arachidonic acid (C20:4), eicosapentaenoic acid (EPA (C20:5), docosenoic acid (C22:1), docosahexaenoic acid (DHA (C22:6), lignoceric acid (C24:0), nervonic acid (C24:1)

### Enzyme activities

Activities of elongase and desaturase enzymes by the FA composition with a range of serum lipids in hepatitis B patients and controls were calculated (Table 6). The elongase, Δ 5 desaturase, and Δ 6 desaturase activities were found lower whereas Δ 9 desaturase activity was found higher in hepatitis B patients in comparison to controls.

**Table 6:** Enzyme activities of hepatitis B patients in comparison to controls

Enzymes	Hepatitis B patients enzyme activity (n = 50)	Control subjects enzyme activity (n = 50)
Elongase	0.48 ± 0.2*	0.69 ± 0.2
Δ-9 desaturase (C16)	0.13 ± 0.03*	0.15 ± 0.09
Δ- 9 desaturase (C18)	1.83 ± 0.7*	1.53 ± 0.5
Δ-5 desaturase	7.1 ± 3.2*	9.8 ± 7.2
Δ-6 desaturase	0.01 ± 0.01*	0.03 ± 0.04

Values are mean ± standard deviation,*shows significant difference from Hepatitis B patients with comparison of healthy controls a *p* < 0.0001 (t-test)

### Fatty acids association with hepatitis B virus

Odds ratios were calculated (Table 7) for both hepatitis B patients and controls by quartile of serum fatty acids. Present study found an increased risk of HBV progression associated positively with increasing levels of myristic, palmitic, stearic, arachidic, palmitoleic, oleic and docosenoic acids. The significant association between serum FA’s and HBV was found when we compared the odds ratio for the highest quartile with the lowest one. For myristic acid odd ratio was found significant as 4.7 (95% CI: 0.2, 212.2; *p* value = < 0.01), for palmitic acid 3.2 (95% CI: 0.1, 121.0; *p* value = < 0.01). On the contrary PUFA was inversely associated with HBV progression; odds ratio for the highest quartile with the lowest one was calculated as linoleic acid 0.1 (95% CI: 0.003, 2.4; *p* value = < 0.01),α-linolenic acid 0.2 (95% CI: 0.01, 5.1; *p* value = 0.03), eicosatrienoic acid 0.2 (95% CI: 0.01, 4.8; *p* value = 0.02), arachidonic acid 0.8 (95% CI: 0.01, 42.6; *p* value = < 0.01), eicosapentaenoic acid 0.3 (95% CI: 0.01, 6.3; *p* value = 0.003) and docosapentaenoic acid odds ratio was 1.2 (95% CI: 0.2, 8.4; *p* value = 0.05).

**Table 7:** Odd ratios for HBV patients (n = 50) and controls (n = 50) according to quartile of serum fatty acids

Fatty acids	Odds ratio (95% confidence interval)					*P* value
	1^st^ Quartile Reference	2^nd^ Quartile	3^rd^ Quartile	4^th^ Quartile	5^th^ Quartile	
C – 14:0	1.00	1.7 (0.1–21.3)	3.3 (0.4–29.0)	3.1 (0.4–25.2)	4.7 (0.2–212.2)	< 0.01
C - 16: 0	1.00	1.4 (0.2–9.6)	1.7 (0.2–12.3)	1.7 (0.2–14.6)	3.2 (0.1–121.0)	< 0.01
C - 18: 0	1.00	1.4 (0.2–10.9)	1.2 (0.1–13.7)	1.1 (0.2–6.6)	2.4 (0.1–93.9)	0.26
C - 20: 0	1.00	1.7 (0.1–24.2)	2.1 (0.4–11.4)	2.2 (0.3–19.8)	3.3 (0.2–118.8)	0.29
C - 16: 1	1.00	3.0 (0.2–96.4)	3.4 (0.2–103)	3.0 (0.03–904)	3.6 (0.2–125.9)	0.11
C - 18: 1	1.00	1.2 (0.1–16.3)	1.9 (0.2–14.8)	2.0 (0.2–19.7)	3.3 (0.1–158.8)	0.15
C - 22: 1	1.00	2.4 (0.3–22.6)	2.7 (0.2–35.8)	2.2 (0.3–16.9)	2.0 (0.03–117)	0.29
C - 18: 2	1.00	0.6 (0.1–4.1)	0.4 (0.02–4.3)	0.2 (0.01–3.6)	0.1 (0.003–2.4)	< 0.01
C - 18: 3	1.00	0.2 (0.01–1.9)	0.5 (0.1–3.4)	0.4 (0.1–2.7)	0.2 (0.01–5.1)	0.03
C - 20: 3	1.00	0.2 (0.02–1.4)	0.2 (0.01–2.6)	0.4 (0.1–2.3)	0.2 (0.01–4.8)	0.02
C - 20: 4	1.00	0.6 (0.1–5.5)	0.8 (0.1–5.4)	0.8 (0.1–6.5)	0.8 (0.01–42.6)	< 0.01
C - 20: 5	1.00	0.5 (0.04–4.6)	0.5 (0.04–4.6)	0.5 (0.1–3.4)	0.3 (0.01–6.3)	0.003
C - 22: 5	1.00	1.0 (0.1–8.2)	1.0 (0.1–8.2)	1.0 (0.01–53)	1.2 (0.2–8.4)	0.05
C - 22: 6	1.00	0.8 (0.1–7.5)	0.7 (0.1–4.8)	0.8 (0.1–5.8)	0.8 (0.01–42.6)	0.25

### Difference biomarkers

**Fig. 4:**
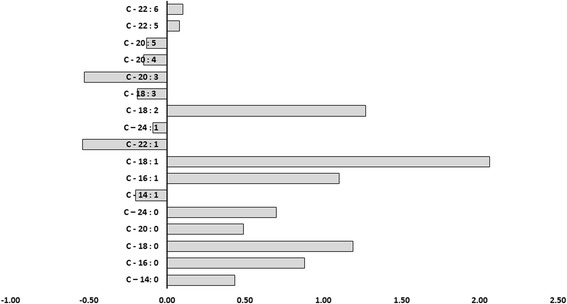
Difference between the average saturated and unsaturated fatty acids concentrations exhibited significant changes (hepatitis B cirrhosis/hepatitis B)

The Fig. 4 shows the plot of difference in the average fatty acids concentrations, which exhibited significant changes between hepatitis B cirrhosis with hepatitis B patients. The hepatitis B cirrhosis patient’s data was used from our previous paper [14]. The increase in nervonic, α-linolenic, eicosatrienoic, arachidonic and eicosapentaenoic acids were observed as potential differentiating biomarkers in hepatitis B in comparison of hepatitis B cirrhosis patients.

## Discussion

HBV cells interact with cholesterol molecules for involving and spreading of infection in the target cells, although binding to the cell surface remains unaffected, which suggested that cholesterol within the viral envelope is required for later step in viral fusion [15]. The lipids have been considered to play an important role in the host immune response mechanism to infections [16]. Lipoproteins can bind a variety of viruses and reduces their toxic effects [17]. The binding of apolipoprotein H (apo H) to the HBsAg with subsequent lowering of the plasma apolipoprotein. The prolonged surge in pro-inflammatory cytokines such as tumor necrosis factor-alpha (TNF-α), interleukin (IL)-1, IL-6, and interferon-alpha (IFN-α) has been linked with dyslipidemia in studies following the chronic state of the HBV-infection. Thus, interaction of HBsAg with apo H and lipidemic effect of cytokines could have contributed to the observed distortions in the various lipid indices of the hepatitis B infected population [18].

Decreased levels of total cholesterol and HDL-C in patients with asymptomatic chronic HBV infection may reduce their antiviral response. In patients with chronic hepatitis B, a high viral load is usually associated with active disease and histological progression in the long run [19]. The detailed mechanism responsible for the HDL-C reducing effect of chronic HBV infection remains to be clear. An enzyme involved in the biotransformation of HDL-C, Lecithin-Cholesterol Acyl Transferase (LCAT), was reported to reduce in patients with advanced chronic liver disease or cirrhosis, probably with the reason of impaired hepatic synthesis [20]. This reduction may also decrease HDL-C and TC in the case of mild hepatic damage that is not reflected.

Chronic HBV infection was associated with the metabolic syndrome that possibly enhances the risk of fibrosis progression in liver cirrhosis and responsible for the development of HCC. If fibrosis continues unopposed, it would disrupt the normal architecture of the liver which alters the normal function of the organ, ultimately leading to pathophysiological damage of the liver [21]. In current study, the hepatitis B patients belongs to the early stage of fibrosis because cirrhosis represents the final stages of fibrosis. When liver function in cirrhotic liver proceeds into decompensated, it is no doubt that liver transplantation is the only effective way to save patients’ lives. The criteria of urgent liver transplantation depend upon the MELD score for non-cirrhotic patients with acute HBV flare up and liver failure. The MELD score was found 20 for hepatitis B patient showed 6.0% estimated mortality risk within 3 months. King College’s criteria was used for the evaluation of liver transplantation necessity, when the level of serum total bilirubin is 17.5 mg/dL, that indicates urgent liver transplantation upon MELD scores ≥35 at beginning and increased in the subsequent 1 to 2 weeks [22].

The percentile provides more information about the relationship of exposure to disease risk than the common method of grouping the data by specific percentiles, e.g. quartiles. It has been reported previously that the percentiles of triacylglycerol, serum fatty acids phospholipid and cholesterol ester provides population-based reference ranges against which the fatty acid status/lipid profile of individuals or groups can be compared. In addition, these reference ranges can be useful to researchers and epidemiologists [23].

It’s been identified in the current study, that the FA metabolic features are associated with HBV infection. The palmitic acid in hepatitis B patients is found significantly elevated and reduced stearic acid which may be due to impaired FA molecular structure elongation. This theory was explained by Moon et al., (2001) [24] after experimental studies on rodents, it is estimated that 90% of the endogenously synthesized stearate occurs through the elongation of palmitate in the endoplasmic reticulum and not through cytosolic fatty acids. The data presented suggest that long chain fatty acyl elongase is a member of the elusive elongation system present in the endoplasmic reticulum of mammals that is likely responsible for elongation of palmitic acid to stearic acid in vivo.

The low level of stearic acid while an increase in oleic acid and higher oleic to stearic acid ratios in hepatitis B patients has shown decreased levels of downstream metabolites arachidic acids; similar results are also reported in non-alcoholic steatohepatitis due to decreased activity of elongase and increased activity of Δ 9 desaturase [25].

The fatty acyl elongases (ELOVL; elongation of very long chain fatty acids) catalyze elongation process of FA. These enzymes are playing an important role in the initial step (condensation of FA) in elongation pathway. The seven family members of fatty acyl elongase (ELOVL1 to ELOVL7) have been identified in humans and mice with the characteristic of specific FA substrate. The ELOVL1, ELOVL3, ELOVL6, and ELOVL7 use SFA and MUFA as substrates, whereas ELOVL2, ELOVL4, and ELOVL5 prefer PUFA as substrates [26]. The elongase enzyme activity was found lower in hepatitis B patients in comparison to controls. The Elovl-6 enzyme elongates saturated FA palmitic to stearic acid [27]. The reduced level of stearic acid indicates the lower activity of the Elovl-6 enzyme. The decreased levels of PUFA in hepatitis patients in comparison to controls, which specifies the reduced activities of ELOVL2, ELOVL4 and ELOVL5 enzymes. The lower level of nervonic acid in hepatitis B cirrhosis patients in comparison of hepatitis B patients indicate the ELOVL1 lower activity in hepatitis B cirrhosis patients, because ELOVL1 production of lignoceric acid and nervonic acid acyl-CoAs is essential for the synthesis of C24 sphingolipids [28]. The nervonic acid is an essential molecule for the growth and maintenance of the brain and peripheral nervous tissue enriched in sphingomyelin, as sphingomyelin is a key constituent of myelin, it is abundant in the white matter of the brain [29].

FA of the n-6 series and n-3 series are essential for mammals. FA in the omega-6 family, linoleic (n-6) and arachidonic (n-6), and those in the omega-3 family, α-linolenic (n-3) must be supplied in human diets. The synthesis pathways of n-3 and n-6 fatty acids compete for the ∆5 and ∆6 desaturases though both enzymes preferentially catalyze the reactions of the n-3 pathway. The α-linolenic can be converted to eicosapentaenoic and docosahexaenoic acid in the mammalian liver by a series of desaturation and elongation reactions and the ∆ 5-desaturation of arachidonic acid generates eicosapentaenoic acid (n-3) [30, 31]. The fatty acid desaturase 1 (FADS1) is also termed as ∆5 desaturase and fatty acid desaturase 2 (FADS2) named as ∆ 6 desaturase, these enzymes catalyzed desaturation of PUFA in the liver, signifying as key enzymes for biosynthesis of long chain PUFA [32]. The reduced level of PUFA (n-3, n-6), is possibly due to lower activities of ∆ 5 desaturase and ∆ 6 desaturase enzymes and higher activity of ∆ 9 desaturase in hepatitis B patients that enumerate impair activity of FADS1 and FADS2.

The nervonic, α-linolenic, eicosatrienoic, arachidonic and eicosapentaenoic acids were higher in hepatitis B patients observed as potential differentiating biomarkers in comparison of hepatitis B cirrhosis patients. The lower level of α-linolenic, eicosatrienoic, arachidonic and eicosapentaenoic in hepatitis B cirrhosis patients indicate ∆5 and ∆6 desaturases was lower in hepatitis B cirrhosis in comparison of hepatitis patients. Arachidonic acid enhances the release of glutamate neurotransmitter, inhibits neurotransmitter uptake, stimulates stress hormone secretion, and enhances synaptic transmission. In the immune system, arachidonic acid can trigger an inflammatory response and increase oxidants in the brain and eicosapentaenoic acid can protect neurons from inflammation and oxidants [33].

The PUFA levels are reduced in hepatitis B patients includes linoleic, α- linolenic, eicosatrienoic, arachidonic, eicosapentaenoic acids furthermore responsible for the decrease in the total PUFA contents and PUFA to SFA ratio in HBV patients. The similar results were reported in alcoholic liver cirrhosis patients, as low levels of PUFA was found which in turn due to changes of Δ-5, Δ-6 and Δ-9 desaturase activity, which may lead to an increased degradation of PUFA due to lipid peroxidation [34].

In the hepatitis B patients, serum docosapentaenoic acid was elevated with downstream metabolite docosahexaenoic acid. The decline in docosahexaenoic acid is potentially important because docosahexaenoic acid is formed in peroxisomes from docosapentaenoic acid. The increase in docosapentaenoic acid along with declines in docosahexaenoic acid proposes the impaired peroxisomal PUFA metabolism [35]. These results provide motivation to forthcoming studies for the role of peroxisomal dysfunction in the pathogenesis of hepatitis B patients.

The palmitic and stearic acid in free form is significantly higher in hepatitis B patients. The FFA contain lipotoxic properties reported in the literature [36]. The saturated FFA’s, palmitic acid and stearic acid are more cytotoxic than monounsaturated FFAs. The foundation of reactive oxygen species through another lipid metabolic pathways, such as modulation of death receptor expression, ceramide synthesis and direct activation of cellular proapoptotic machinery are putative mechanisms by which lipoapoptosis is thought to occur [37], which promote the hepatic lipotoxicity in hepatitis B patients.

The eicosapentaenoic and docosahexaenoic acid is reduced in hepatitis B patients. The FA’s immunomodulating activities are also reported in the literature [38]. The n-3 fatty acids contained the most effective immunomodulatory activities; eicosapentaenoic and docosahexaenoic acid are highly biological active than α-linolenic acid [39]. The impaired immunity and deficiency of immunomodulating nutrients reactivity may be linked with hepatitis B antigen. It is possible that management of n-3 fatty acid decrease infection frequency and increases recovery of liver function in Hepatitis B patients suffering from hepatitis B infection.

Host immune factors play essential roles in the outcome of HBV infection. Thus, ineffective immune response against HBV may result in persistent virus replications and liver necro-inflammations. Cytokine balance was shown to be an important immune characteristic in the development and progression of hepatitis B, as well as in an effective antiviral immunity. The cytokines are the primary causes of inflammation and mediates liver injury after HBV infection, the roles of various cytokines [including T helper type 1 cells (Th1), Th2, Th17, regulatory T cells (Treg)-related cytokines] in different phases of HBV infection and cytokine-related mechanisms for impaired viral control and liver damage during HBV infection. Th17 cells are a newly identified subset of T helper cells and are closely related to the progression of HBV disease. Research interest in these cells has indicated that patients with chronic HBV infection were found to have significantly elevated Th17 cell frequency and Th17-secreted cytokines, including interleukin IL-17A, IL-21, and IL-22, and it was proposed that these pro-inflammatory effectors may perform a vital function in pathogenesis of prolonged hepatitis B infection [40].

The present study supports an inverse association of PUFA with HBV progression. The lipids emulsion has the capability to transform the immune mechanism. The n-3fatty acids are known for their capability to modify leukocyte activity, amend lipid-mediator generation and to modulate cytokine release. The declining of infectious phase was reported after the initiation of n-3 FA’s a by enteral nutrition to hepatic trauma patients [41]. It is also suggested by the current study that use of n-3 and n-6 FA’s significantly decreases the rate and complications of disease when meaningfully added in the diet of hepatitis B patients.

## Conclusion

Hypolipidemia is observed in hepatitis B patients due to impaired liver function. The increased level of SFA and decreased the level of PUFA were detected in hepatitis B patients due to altered activities of enzyme elongase, ∆5, ∆6 and ∆9-desaturase with impaired PUFA metabolism and non-enzymatic oxidation.

### Limitations of the study

In present study numbers of samples are not large enough, though ideally larger group of subjects are required to delineate the role of lipid synthesis across the HBV spectrum.

## Additional files

Additional file 1: Figure S1. Chromatogram of fatty acids standards. Figure S2 (a). Chromatogram of serum total fatty acids of hepatitis B patients. (b). Chromatogram of serum total fatty acids of control subject. Figure S3 (a). Chromatogram of serum free fatty acids of hepatitis B patient. (b). Chromatogram of serum free fatty acids of control subject. (DOCX 1378 kb)

Additional file 2: Lipid profile and serum fatty acid composition data of hepatitis B patients and controls. (XLSX 57 kb)

## Abbreviations

CVD: Cardiovascular diseases
D5D: ∆5-desaturase
D6D: ∆6-desaturase
D9D: ∆9-desaturase
FA: Fatty acids
FADS: Fatty acid desaturase
FAS: Fatty-acid synthase
FID: Flame ionization detector
GC: Gas chromatography
HBV: Hepatitis B virus
HDL-C: High density lipoprotein-cholesterol
LDL-C: Low density lipoprotein-cholesterol
MUFA: Monounsaturated fatty acid
NAFLD: Nonalcoholic fatty liver disease
PUFA: Poly unsaturated fatty acid
SCD: Stearoyl-CoA desaturase
SFA: Saturated fatty acid
TAG: Triacylglycerol
VLDL-C: very low density lipoprotein-cholesterol
