# Supplementary material for: Serum lipids as an indicator for the alteration of liver function in patients with hepatitis B
Source: Lipids Health Dis. 2018 Mar 5;17:36. doi: 10.1186/s12944-018-0683-y (PMC5838863; doi:10.1186/s12944-018-0683-y)
Supplement: Supplementary file 1 — Figure S1. Chromatogram of fatty acids standards. Figure S2 (a). Chromatogram of serum total fatty acids of hepatitis B patients. (b). Chromatogram of serum total fatty acids of control subject. Figure S3 (a). Chromatogram of serum free fatty acids of hepatitis B patient. (b). Chromatogram of serum free fatty acids of control subject. (DOCX 1378 kb) [file 12944_2018_683_MOESM2_ESM.docx]

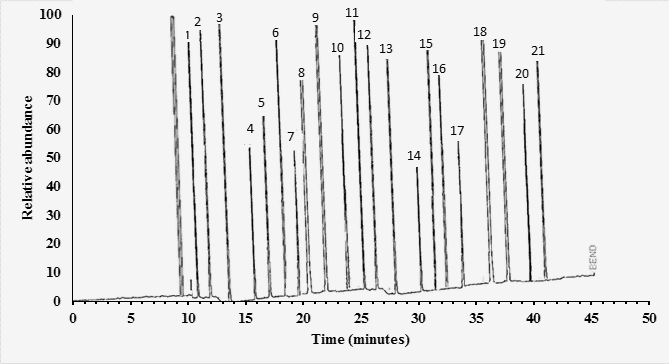


**1**, C- 8:0; **2**,C – 10:0; **3**, C – 12:0; **4**, C – 14:0; **5**, C – 14: 1; **6**, C – 15: 0; **7**, C – 16:0; **8**, C – 16:1; **9**, C – 18:0; **10**, C – 18: 1; **11**, C– 18:2; **12**, C – 20:0; **13**, C – 18:3; **14**, C – 20:3; **15**, C – 22:1; **16**, C – 20:4; **17**, C – 20:5; **18**, C – 22:5; **19**, C – 22:6; **20**, C – 24:0; **21**, C – 24:1.

Figure 1. Chromatogram of fatty acids standards


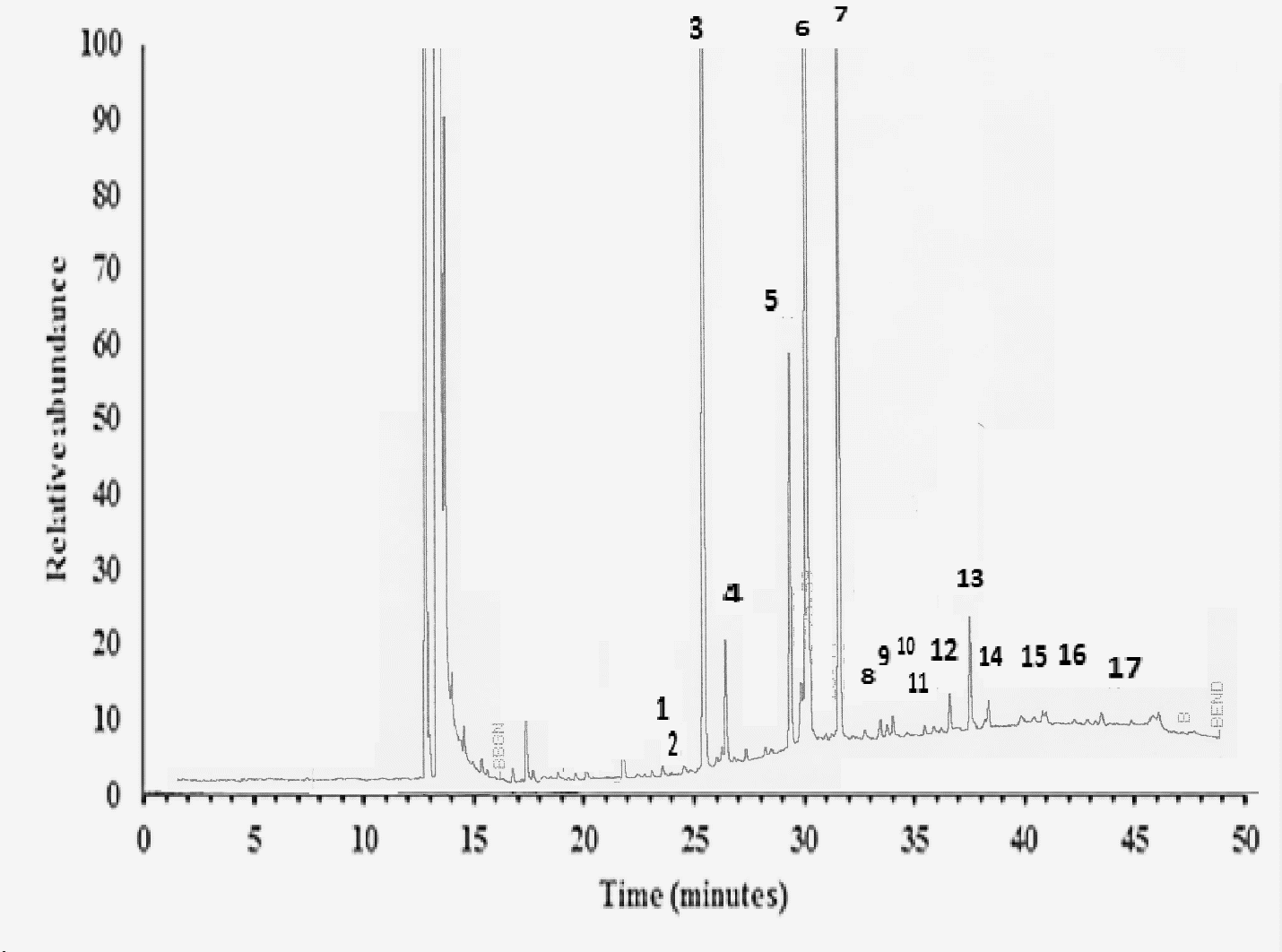


**1,** C – 14:0; **2,** C – 14: 1; **3,** C – 16:0; **4,** C – 16:1; **5,** C – 18:0; **6,** C – 18: 1; **7,** C– 18:2, **8,** C – 20:0; **9,** C – 18:3; **10,** C – 20:3; **11,** C – 22:1; **12,** C – 20:4; **13,** C – 20:5; **14,** C – 22:5; **15,** C – 22:6; **16,** C – 24:0; **17,** C – 24:1.

Figure 2 (a). Chromatogram of serum total fatty acids of hepatitis B patients


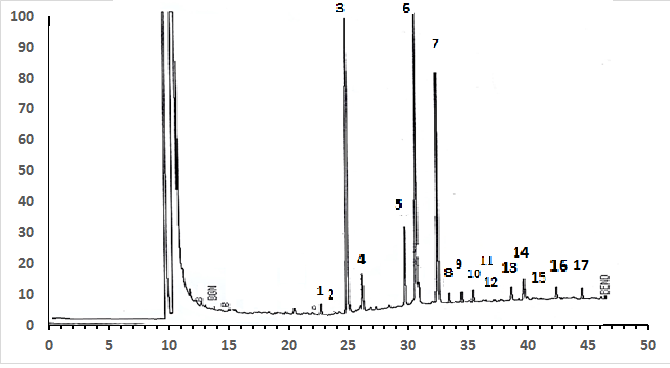


**1,** C – 14:0; **2,** C – 14: 1; **3,** C – 16:0; **4,** C – 16:1; **5,** C – 18:0; **6,** C – 18: 1; **7,** C– 18:2, **8,** C – 20:0; **9,** C – 18:3; **10,** C – 20:3; **11,** C – 22:1; **12,** C – 20:4; **13,** C – 20:5; **14,** C – 22:5; **15,** C – 22:6; **16,** C – 24:0; **17,** C – 24:1.

Figure 2 (b). Chromatogram of serum total fatty acids of control subject


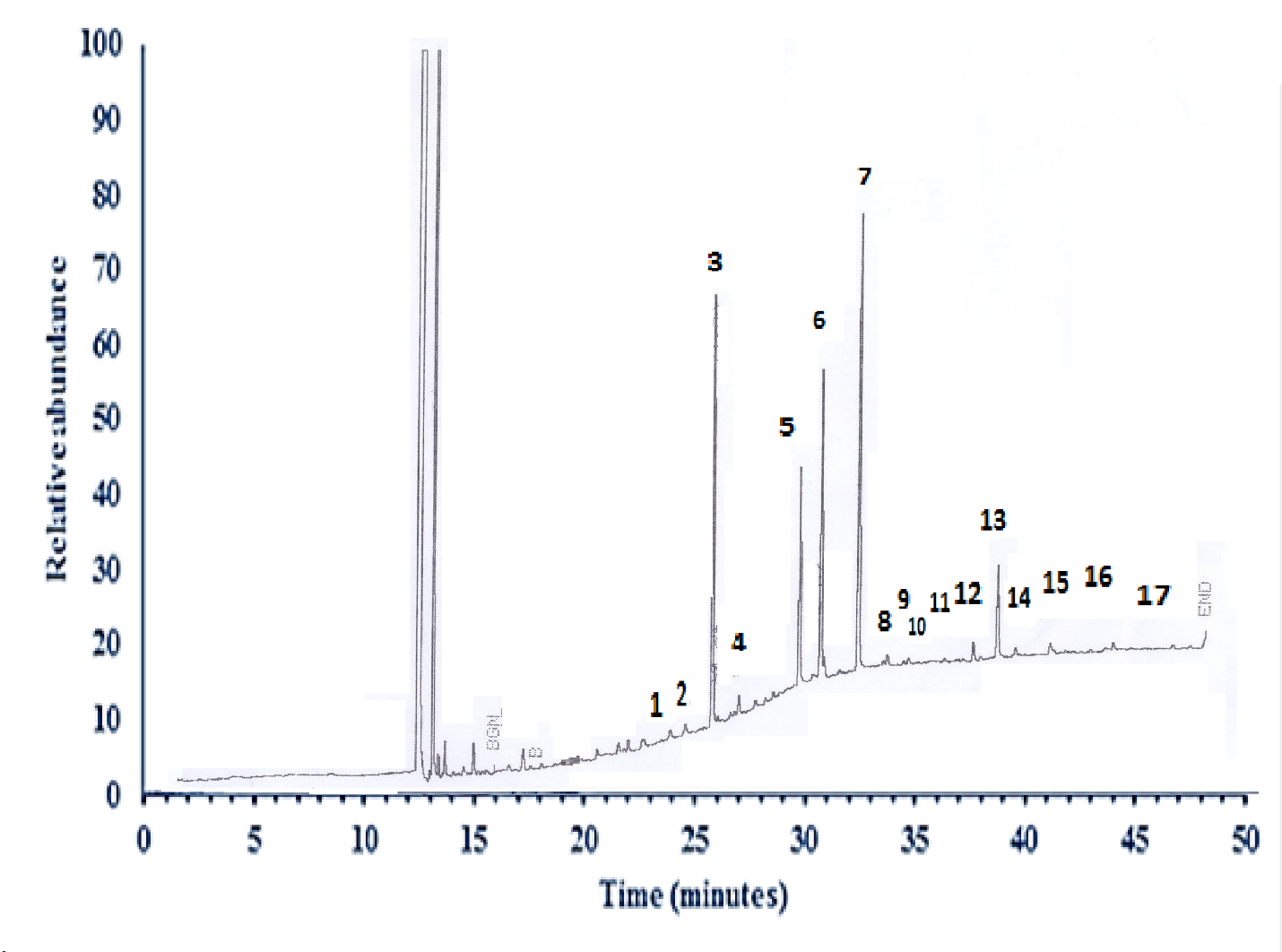


**1,** C – 14:0; **2,** C – 14: 1; **3,** C – 16:0; **4,** C – 16:1; **5,** C – 18:0; **6,** C – 18: 1; **7,** C– 18:2, **8,** C – 20:0; **9,** C – 18:3; **10,** C – 20:3; **11,** C – 22:1; **12,** C – 20:4; **13,** C – 20:5; **14,** C – 22:5; **15,** C – 22:6; **16,** C – 24:0; **17,** C – 24:1.

Figure 3 (a). Chromatogram of serum free fatty acids of hepatitis B patient


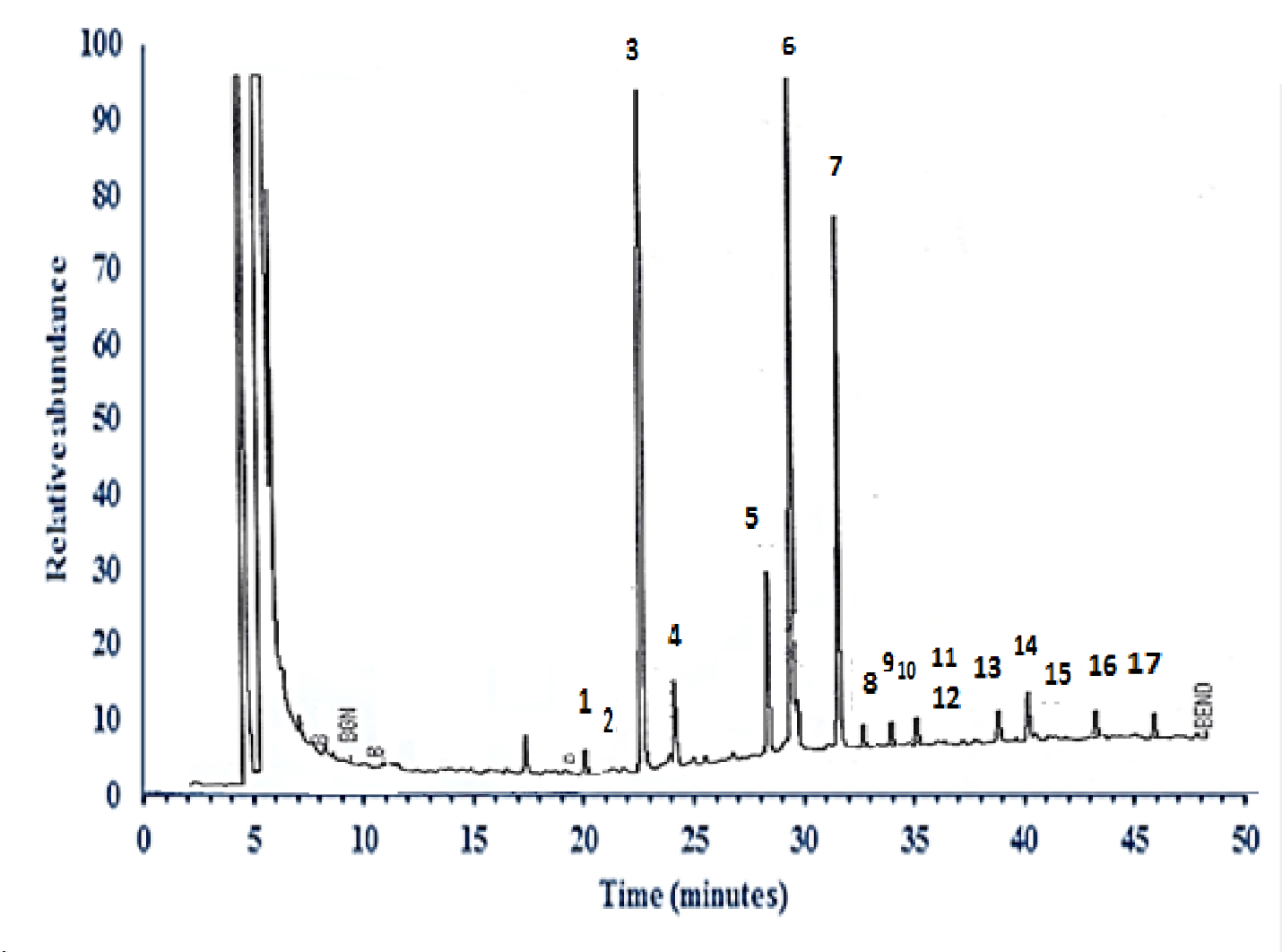


**1,** C – 14:0; **2,** C – 14: 1; **3,** C – 16:0; **4,** C – 16:1; **5,** C – 18:0; **6,** C – 18: 1; **7,** C– 18:2, **8,** C – 20:0; **9,** C – 18:3; **10,** C – 20:3; **11,** C – 22:1; **12,** C – 20:4; **13,** C – 20:5; **14,** C – 22:5; **15,** C – 22:6; **16,** C – 24:0; **17,** C – 24:1.

Figure 3 (b). Chromatogram of serum free fatty acids of control subject.
